# A Diverse View of Science to Catalyse Change†

**DOI:** 10.1002/anie.202009834

**Published:** 2020-08-17

**Authors:** César A. Urbina‐Blanco, Safia Z. Jilani, Isaiah R. Speight, Michael J. Bojdys, Tomislav Friščić, J. Fraser Stoddart, Toby L. Nelson, James Mack, Renã A. S. Robinson, Emanuel A. Waddell, Jodie L. Lutkenhaus, Murrell Godfrey, Martine I. Abboud, Stephen O. Aderinto, Damilola Aderohunmu, Lučka Bibič, João Borges, Vy M. Dong, Lori Ferrins, Fun Man Fung, Torsten John, Felicia P. L. Lim, Sarah L. Masters, Dickson Mambwe, Pall Thordarson, Maria‐Magdalena Titirici, Gabriela D. Tormet‐González, Miriam M. Unterlass, Austin Wadle, Vivian W.‐W. Yam, Ying‐Wei Yang

**Affiliations:** ^1^ Laboratory for Chemical Technology Ghent University Ghent B-9052 Belgium; ^2^ Department of Chemistry Georgetown University Washington DC 20057 USA; ^3^ Department of Chemistry Vanderbilt University Nashville TN 37235 USA; ^4^ Department of Chemistry, King's College, London (UK) and Department of Chemistry Humboldt-Universität zu Berlin Berlin 12489 Germany; ^5^ Department of Chemistry McGill University Montréal QC H3A 0B8 Canada; ^6^ Department of Chemistry Northwestern University Evanston IL 60208 USA; ^7^ Institute for Molecular Design and Synthesis Tianjin University Tianjin 300072 People's Republic of China; ^8^ School of Chemistry University of New South Wales Sydney NSW 2052 Australia; ^9^ Department of Chemistry Oklahoma State University Stillwater OK 74078 USA; ^10^ Department of Chemistry University of Cincinnati Cincinnati OH 45221 USA; ^11^ Department of Chemistry University of Alabama in Huntsville Huntsville AL 35899 USA; ^12^ Artie McFerrin Department of Chemical Engineering Texas A&M University College Station TX 77843 USA; ^13^ Department of Chemistry The University of Mississippi University MS 38677 USA; ^14^ Department of Chemistry University of Oxford Chemistry Research Laboratory Oxford OX1 3TA UK; ^15^ Department of Chemistry The University of Sheffield Sheffield S3 7HF UK; ^16^ Department of Chemistry Covenant University, CST, Canaanland Ota Ogun State Nigeria; ^17^ School of Pharmacy University of East Anglia Norwich NR4 7TJ UK; ^18^ Department of Chemistry CICECO—Aveiro Institute of Materials University of Aveiro Campus Universitário de Santiago Aveiro 3810-193 Portugal; ^19^ Department of Chemistry University of California Irvine CA 92697 USA; ^20^ Department of Chemistry and Chemical Biology Northeastern University Boston MA 02115 USA; ^21^ Department of Chemistry National University of Singapore Singapore 117543 Singapore; ^22^ Leibniz Institute of Surface Engineering (IOM) Leipzig 04318 Germany; ^23^ School of Pharmacy Monash University Malaysia Selangor Darul Ehsan 47500 Malaysia; ^24^ School of Physical and Chemical Sciences University of Canterbury Christchurch 8140 New Zealand; ^25^ Department of Chemistry University of Cape Town Rondebosch Capetown 7701 South Africa; ^26^ School of Chemistry The Australian Centre for Nanomedicine and the ARC Centre of Excellence in Convergent Bio-Nano Science and Technology The University of New South Wales Sydney NSW 2052 Australia; ^27^ Department of Chemical Engineering Imperial College London South Kensington Campus London SW7 2AZ UK; ^28^ Institute of Chemistry University of Campinas (UNICAMP) Campinas SP 13083-970 Brazil; ^29^ Institute of Materials Chemistry Technische Universität Wien Vienna 1060 Austria; ^30^ Department of Civil & Environmental Engineering Duke University Pratt School of Engineering Durham NC 27708 USA; ^31^ Institute of Molecular Functional Materials and Department of Chemistry The University of Hong Kong Hong Kong People's Republic of China; ^32^ State Key Laboratory of Inorganic Synthesis and Preparative Chemistry International Joint Research Laboratory of Nano-Micro Architecture Chemistry (NMAC) College of Chemistry Jilin University Changchun 130012 People's Republic of China

**Keywords:** diversity, equity, excellence, inclusion

## Abstract

**Valuing diversity** leads to scientific excellence, the progress of science and most importantly, it is simply the right thing to do. We can value diversity not only in words, but also in actions.

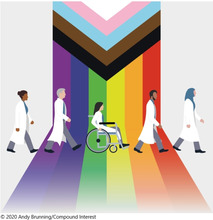

From the structure of DNA,^1^ to computer science,^2^ and space‐station batteries,^3^ several key scientific discoveries that enhance our lives today, were made by marginalized scientists. These three scientists, Rosalind E. Franklin, Alan M. Turing and Olga D. González‐Sanabria, did not conform to the cultural expectations of how scientists should look and behave. Unfortunately, marginalized scientists are often viewed as just a resource rather than the lifeblood that constitutes science itself. We need to embrace scientists from all walks of life and corners of the globe; this will also mean that nobody is excluded from tackling the life‐threatening societal challenges that lie ahead. An awareness of science policy is essential to safeguarding our future.

Science policy deals with creating the framework and codes of conduct that determine how science can best serve society.^4^, ^5^, ^6^ Discussions around science policy are often accompanied by anecdotes of “good” and “bad” practices regarding the merits of diversity and inclusion. Excellence and truth, which flow inexorably from diversity and inclusion, are the bedrocks upon which science should influence political and economic outcomes. A vital area of science policy is to support the professional development of marginalized scientists, an objective that must be acted upon by scientific leaders and communicators.

## Diversity 101

To paraphrase Zimmerman and Anastas,^7^ on the topic of green chemistry, if people are confused about what diversity, equity and inclusion (DEI) are, it is difficult to imagine that from confusion will arise a clear path on how to implement them. If we want to achieve DEI in science, we need to be clear about the definitions of the following key terms.

**Diversity**. The ways in which people differ, encompassing all the characteristics that make one individual or group distinctive.^8^ The dimensions of diversity include, but are not limited to (i) ethnic or national origins, skin colour or nationality, (ii) gender, gender identity and gender expression, (iii) sexual orientation, (iv) background (socio‐economic status, immigration status or class), (v) religion or belief (including absence of belief), (vi) civil or marital status, (vii) pregnancy and maternity, paternity, parental leave and (viii) age and (ix) disability.^9^

**Equity**. The fair treatment, access and opportunity that leads to the advancement of all peoples. Equity is about striving to identify and remove barriers that have prevented the full participation of some groups. Improving equity means increasing justice and fairness within the processes of institutions or systems, as well as communication and sharing of resources. Addressing issues of equity require a deep understanding of the sources of disparity in our society.^10^

**Inclusion**. The act of creating an environment in which any individual or group feels (i) welcomed, (ii) safe, (iii) supported, (iv) respected and (v) valued to participate. An inclusive and welcoming culture embraces differences and offers respect in words and actions to all people. It is important to note that while an inclusive group is by definition diverse, a diverse group is not always inclusive. Increasingly, recognition of implicit bias helps organizations to be constructive about addressing issues of inclusion.^10^

**Implicit bias**. People are not neutral in judgement and behaviour, but instead have experience‐based associations and preferences or aversions without being consciously aware of them.^11^

**Microaggressions**. These are often manifestations of implicit bias, typically in the form of comments or actions.^12^

**Marginalized scientists**. Scientists who are at the periphery of social, economic and scientific discussions.

The reason marginalized scientists leave science, technology, engineering and mathematics (STEM) is not an accident. It results from the historic expectations of how a scientist should be perceived.^13^ The pursuit of equity will dismantle these beliefs, driving policy development and creating equal access to positions of leadership and opportunities for all.

This article is a message for (i) current and future scientists, (ii) students, mentors and educators, (iii) science communicators, (iv) publishers and (v) science policy makers. It has two purposes: (1) Provide marginalized scientists and their allies a space to talk about their approach towards scientific advancement, mentorship and how to challenge systemic injustice and (2) Provide actionable advice to implement equity in academia and related businesses and organizations.

### Identifying and quantifying inequity

Science can only expand the research questions and problems defined as important with a broad pool of life experiences and knowledge. Non‐diverse academic environments are closed communities that reinforce traditional stereotypes of who gets to be a scientist. This situation is analogous to the political science phenomena known as “echo chambers”.^14^ Each country has its own demographics, and consequently the make‐up of marginalized populations may differ. Most well‐represented scientists—that means scientists that conform to the cultural expectations of how scientists should look and behave—do not know or understand the challenges that exist for marginalized scientists. The first step towards beginning to understand these challenges is to listen to marginalized scientists. We must then follow by collecting reliable data, informed by the individual experiences of marginalized scientists.^15^, ^16^

For example, in the UK, a 2018 report by the Royal Society of Chemistry (RSC) noted that the percentage of students from minority groups falls from 26 % at the undergraduate level to 14 % at the postgraduate level.^17^ Unfortunately, this study was not able to show the ethnicity data for staff in higher‐education settings. This incomplete dataset highlights the need for transparent and consistent reporting of DEI data from universities. The RSC also shared that the percentage of minority ethnic chemical scientists in academia appears to drop significantly with increasing career stage.^17^ Meanwhile, in the US, a study by *C&EN* found that 12.3 % of the US population is Black, yet only 1.6 % of chemistry professors at the top 50 US universities are Black.^18^

Mapping the diversity landscape of academia across hierarchies is vital to understanding the severity of the underrepresentation of marginalized scientists. This data should be collected and reported on a regular basis so that progress can be monitored transparently. This information gathering will give organizations a quantitative perspective of diversity in their communities, and provide context to create equitable policies and practices.

### Supporting marginalized scientists

Discrimination and lack of social connections in the scientific community have a negative impact on the experiences and performance of marginalized scientists,^19^, ^20^, ^21^ ranging from poor physical and mental health, to low self‐esteem.^22^, ^23^, ^24^ The psychological cost of not feeling socially or professionally connected is impactful, persistent and has a similar effect as physical pain.^24^, ^25^ Regardless of minority status, marginalized populations experience a higher amount of stress.^26^

Every member of the scientific community has a duty to act and create support structures that promote the career development of marginalized scientists. Below are some examples of specific support systems, and how they play a key role in a marginalized scientist's career.

**Mentorship**. Supporting the personal and professional growth, development, and success of scientists through the provision of career and mental‐health advice.^27^ Mentorship has an overall positive effect on retention and career success of mentees across STEM disciplines.^27^ Despite current efforts in DEI, however, marginalized individuals enrolled in STEM degree programs typically receive less mentorship than their well‐represented peers.^28^, ^29^ Research has shown that marginalized scientists already dedicate more hours of service engaging in invisible work, including mentorship, than their peers.^30^, ^31^ This imbalance reduces their available time to perform tasks that are deemed more valuable for career progression. Mentoring marginalized scientists should also be the responsibility of well‐represented scientists.

**Online peer communities**. Communities such as #ScienceTwitter are free resources to build connections, learn about career opportunities, and share expert advice.^32^ These platforms can increase the visibility and reach of scientific work.^33^ Scientists can increase their visibility and use their platform to promote marginalized colleagues.

**Financial support**. The barriers for marginalized scientists pursuing and engaging in scientific careers can be reduced through financial support.^34^ Scientists and scientific organizations need to create and promote equitable financial aid opportunities that support marginalized scientists in career development and be mindful of the costs of participating in networking events.

**Effective inclusion and diversity support**. These systems can identify, and address, the negative experiences of marginalized researchers; they must be approachable, trustworthy and accountable. Research suggests that such support is best provided through independent and impartial structures.^27^

**Recognizing the work of marginalized scientists**. It is crucial that the achievements of marginalized scientists be valued, respected and credited appropriately.^35^, ^36^ This recognition involves (i) reading their work, (ii) engaging in their discoveries, (iii) cooperating in joint research projects, (iv) citing their work and (v) nominating them for leadership positions and awards.

### Expanding and redefining excellence

Excellence in science is often equated to fundamental discoveries with broad societal impact. The conventional view of excellence was historically shaped within non‐diverse communities that celebrate heroes of science like Isaac Newton, Thomas Edison and Albert Einstein as pop‐culture icons—geniuses isolated from societal context.^37^ This narrow perception of excellence results in funnelling of resources into the hands of already recognized, established and well‐represented scientists—the perceived heroes of tomorrow. Further, it limits the progress of science and the development of fundamentally new ideas, and interdisciplinary fields of investigation.^38^

Diversity in science has helped to bring forward advances in areas that the well‐represented cannot fathom, because they do not share the problems and perspectives of marginalized scientists. Furthermore, the technical and societal problems that marginalized scientists value are not weighted equally. It is, not only, that well‐represented scientists have a narrower conception of what constitutes excellence, but also many of them will fail to attain the level of excellence that the achievements of marginalized scientists already have in contemporary society.

If we want to renew our understanding of excellence, we must also renew the composition of the bodies that define it. This renewal could be achieved through the tenure and promotion process. In order for the promotion process to be equitable, all the achievements of scientists in research, teaching, and service must be included in the redefinition of excellence.^39^

Academics should care about DEI because marginalized scientists matter. Academia has been slower to embrace diversity than the private sector where diversity has been linked to the financial bottom line, in that the more diverse the corporation, the more valuable and profitable is the company.^40^ A broad understanding of excellence embraces the diversity of the creators and beneficiaries of science. As institutions redefine excellence to include all, the benefits for all will be tremendous.^40^, ^41^

### Inclusion in the publishing space

Scientific communication throughout the mass media and academic outlets remains the fundamental pillar of the relationship between scientists and society.^42^ Participants in the publishing process, however, do not yet universally reflect the diversity of the scientific community, which itself does not reflect the diversity of society as a whole.^43^ This lack of diversity reduces the participation of marginalized groups when it comes to publishing. Their inclusion will not occur until stakeholders from all parts of the scientific community are represented at all levels of the publishing process. This change means: (i) shaping journal policies, (ii) influencing daily operations, (iii) choosing reviewers, (iv) giving guidance to editorial staff and (v) hiring more diverse teams. Marginalized scientists need to play leadership roles in the establishment of advisory and editorial boards within publishing houses.

Journals can create a more equitable and trustworthy publishing process by stating their mission initiatives clearly and making direct statements addressing any kind of bias against marginalized groups. These statements should be updated annually and be supported by data analysis on the diversity of (i) frontline editorial teams, (ii) reviewers, and (iii) authors both of submitted manuscripts and accepted articles. Given this transparent information, publishers can identify biases and take steps to eliminate them. A larger and equitable talent pool would also unburden the marginalized scientists who are currently stretched thin across editorial positions.

### Conclusion

The uptake of DEI support structures has started to address shortcomings, and we see an upward—but often anecdotal—trend in the inclusion of some marginalized groups in STEM. These efforts, however, focus on dealing with the consequences, rather than eliminating systemic discrimination and implicit bias in academia.^44^ All scientists can contribute to reducing the impact of implicit bias by accepting, learning, and identifying their own biases through active and continuous self‐assessment. For example, Project Implicit, a non‐profit organization, has developed a set of online tools for understanding attitudes, stereotypes and other hidden biases that influence perception, judgment and action.^45^

Reducing the inequalities in STEM requires a data‐based, holistic approach to DEI. We all need to become advocates of marginalized scientists and give them equitable opportunities to advance their careers because it is ultimately the right thing to do. The result will not only be a broader pool of future talents, but also an unprecedented level of excellence that a more colorful and inclusive scientific community can attain.

We have collected statements from scientists that come from all walks of life to share how they value DEI initiatives (https://chemistrycommunity.nature.com/channels/diverse‐views‐in‐science). These statements contain individual calls to action, as well as broader advice to the younger scientists. We hope that you find them interesting and, in the words of Michael Polanyi,^46^ to use them for a “coordination by mutual adjustment of independent initiatives.” Let us use these statements to learn from each other as we do in science.

## Conflict of interest

The authors declare no conflict of interest.
